# TiO_2_ Simultaneous Enrichment, On-Line Deglycosylation, and Sequential Analysis of Glyco- and Phosphopeptides

**DOI:** 10.3389/fchem.2021.703176

**Published:** 2021-08-11

**Authors:** Cheng Chen, Xiaofei Zhang, Xuefang Dong, Han Zhou, Xiuling Li, Xinmiao Liang

**Affiliations:** ^1^ Key Laboratory of Separation Science for Analytical Chemistry, Dalian Institute of Chemical Physics, Chinese Academy of Sciences, Dalian, China; ^2^ University of Chinese Academy of Sciences, Beijing, China; ^3^ Ganjiang Chinese Medicine Innovation Center, Nanchang, China

**Keywords:** simultaneous enrichment, on-line deglycosylation, sequential elution, phosphopeptides, glycopeptides, TiO_2_

## Abstract

Reversible protein glycosylation and phosphorylation tightly modulate important cellular processes and are closely involved in pathological processes in a crosstalk dependent manner. Because of their significance and low abundances of glyco- and phosphopeptides, several strategies have been developed to simultaneously enrich and co-elute glyco- and phosphopeptides. However, the co-existence of deglycosylated peptides and phosphopeptides aggravates the mass spectrometry analysis. Herein we developed a novel strategy to analyze glyco- and phosphopeptides based on simultaneous enrichment with TiO_2_, on-line deglycosylation and collection of deglycosylated peptides, and subsequent elution of phosphopeptides. To optimize on-line deglycosylation conditions, the solution pH, buffer types and concentrations, and deglycosylation time were investigated. The application of this novel strategy to 100 μg mouse brain resulted in 355 glycopeptides and 1,975 phosphopeptides, which were 2.5 and 1.4 folds of those enriched with the reported method. This study will expand the application of TiO_2_ and may shed light on simultaneously monitoring protein multiple post-translational modifications.

## Introduction

Protein glycosylation and phosphorylation are two of the most ubiquitous and important post-translational modifications (PTMs) and they play vital roles in regulating a variety of physiological and pathological processes. These two types of PTMs rarely work alone but interplay in a crosstalk dependent manner. Increasing lines of evidence indicate that the crosstalk between protein glycosylation and phosphorylation is involved in many important biological events (Hart et al., 2011) and their abnormalities are closely associated with many serious diseases (Liu et al., 2002; Takeda et al., 2015; Ma et al., 2017; Zhang et al., 2017). For example, reciprocal protein glycosylation and phosphorylation co-regulate nutrient sensing, neural development, and cell cycle (Hart et al., 2011); the hyperphosphorylation of tau protein is triggered by its abnormal N-linked glycosylation, which is key to Alzheimer’s disease (Losev et al., 2021). Thus simultaneous monitoring these two PTMs and elucidating of their crosstalk in biological samples, especially for precious and trace of biological samples, have pathological and clinical significance.

**SCHEME 1:**
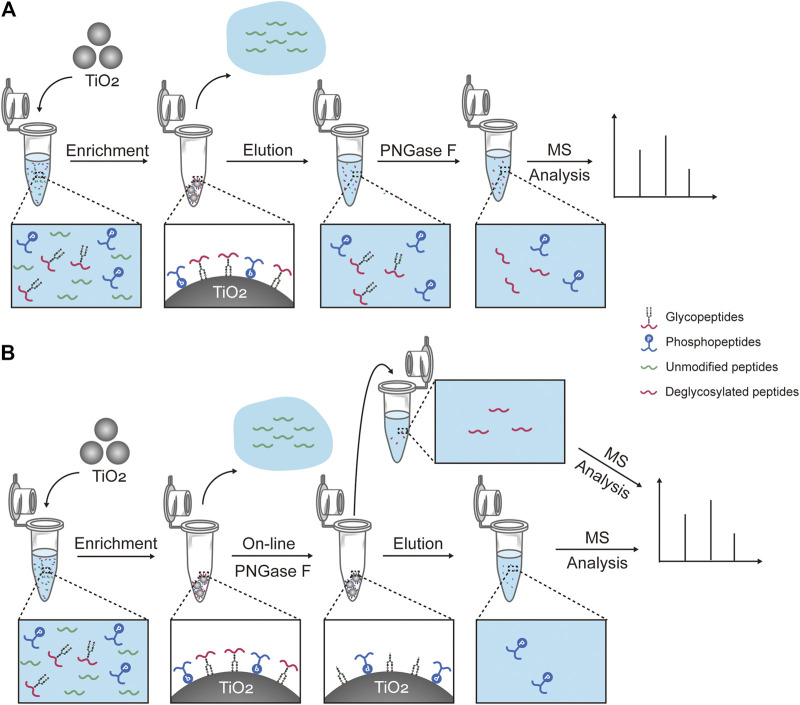
Workflows for capturing and treatment of glyco- and phosphopeptides prior to the mass spectrometry analysis. **(A)** Workflow of one of the reported methods, which consists of the simultaneous enrichment with TiO_2_, the co-elution, and the deglycosylation with PNGase F (hereinafter abbreviated to “reported method”). **(B)** Workflow of our strategy, which consists of the simultaneous enrichment with TiO_2_, the on-line deglycosylation with PNGase F, and the sequential elution.

In the past decade, PTM proteomics has developed rapidly, benefiting from advances of mass spectrometry (MS) technology and improvement of enrichment strategies. However, it remains challenging to simultaneously analyze glyco- and phosphopeptides, due to their low abundances and the high complexity of biological samples. To date, several materials have been developed for simultaneous enrichment of glyco- and phosphopeptides, including metal oxide affinity chromatography- (MOAC-) based materials (Xu et al., 2016; Xu et al., 2017; Sun et al., 2019), immobilized metal ion affinity chromatography- (IMAC-) based materials (Melo-Braga et al., 2014; Zou et al., 2017; Cho et al., 2019; Wang et al., 2019), and hydrogen bond-based polymer material (Lu et al., 2020). As the representative of MOAC-based materials, TiO_2_ is the most commonly used for its excellent robustness (Peng et al., 2017), reproducibility (Sun et al., 2019), and commercial availability. The affinity of TiO_2_ to glycopeptides is based on ligand-exchange and hydrophilic interactions between TiO_2_ and saccharides (Sheng et al., 2013) and binding of TiO_2_ toward phosphopeptides is based on Lewis acid-base interaction between TiO_2_ and phosphate groups (Yan and Deng 2019). In classical TiO_2_ simultaneous enrichment cases (Scheme 1A), the captured glyco- and phosphopeptides are co-eluted (Hu et al., 2018; Palmisano et al., 2012a) and undergo an enzymatic deglycosylation treatment for glycosylation sites identification (Deeb et al., 2014). However, the co-existence of deglycosylated peptides and phosphopeptides will increase the burden of further MS analysis. To reduce the complexity of samples, the two-dimensional enrichment is often employed to address this issue (Melo-Braga et al., 2015), but additional processing steps may lead to a low recovery of targets. Besides, the co-existent phosphopeptides can be hydrolyzed under alkaline deglycosylation conditions (Thompson et al., 2003), and the desalting procedure after deglycosylation will aggravate the loss of PTM-peptides.

Herein, we developed a novel strategy to simultaneously enrich and sequentially analyze glyco- and phosphopeptides, which consists of the simultaneous enrichment of glyco- and phosphopeptides with TiO_2_ and the on-line deglycosylation to obtain deglycosylated peptides and sequential elution of phosphopeptides. The on-line deglycosylation is key to the success of this strategy. Thus, some key factors of the on-line deglycosylation were investigated and optimized, such as solution pH, buffer concentrations, and deglycosylation time. This work will have a great potential in the simultaneous analysis of the protein glycosylation and other multiple PTMs.

## Experiments

### Reagents and Standards

HPLC-grade acetonitrile (ACN), urea, ammonium hydroxide (NH_3_·H_2_O), DL-dithiothreitol (DTT), iodoacetamide (IAA), ammonium formate (HCOONH_4_), ammonium acetate (NH_4_OAc), ammonium bicarbonate (NH_4_HCO_3_), formic acid (FA), acetic acid, glycolic acid, [Glu1]-Fibrinopeptide B human (GFB) (internal standard), bovine fetuin (standard glycoprotein), α-casein (standard phosphoprotein), and trypsin were purchased from Sigma-Aldrich (St Louis, MO, United States). Standard phosphopeptide (with sequence of HS^*^PIAPSSPSPK) was synthesized by Qiangyao Biotechnology Co., Ltd. (Shanghai, China). Trifluoroacetic acid (TFA), acetone, and ethyl alcohol were purchased from Shanghai Macklin Biochemical Co., Ltd. (Shanghai, China). PNGase F was purchased from New England Biolabs (Ipswich, MA, United States). Radioimmunoprecipitation (RIPA) lysis buffer and bicinchoninic acid (BCA) protein assay kit were purchased from Beyotime Biotechnology (Shanghai, China). GELoader was purchased from Eppendorf (Hamburg, Germany). TiO_2_ was purchased from GL Sciences (Tokyo, Japan). C18HC material was purchased from ACCHROM (Wenling, China). Mouse brains were provided by Dalian Medical University (Dalian, China). Pure water was purified with a Milli-Q system (Millipore, Milford, MA, United States).

### Instruments

The peptide samples and TiO_2_ were mixed in a thermomixer (Qianjun, Shanghai, China). TiO_2_ was separated from the mixture by centrifuge (Merck, Milford, MA, United States). A Labconco CentriVap system (Labconco, Kansas, MO, United States) was applied to dry samples in specific steps. Determination of protein concentrations was by a microplate reader (Thermo Scientific, San Jose, CA, United States). The qualitative analysis of the standard protein digests was conducted on a nano electrospray ionization quadrupole time-of-flight mass spectrometer (ESI-Q-TOF MS) (Waters, Manchester, United Kingdom). The qualitative analysis of the protein digests extracted from the mouse brains was performed using an Orbitrap Eclipse Tribrid mass spectrometer and a Dionex UltiMate 3000 rapid separation liquid chromatography (RSLC) system (Thermo Scientific, San Jose, CA, United States).

### Protein Extraction

A mouse brain tissue was cleaned and cut into pieces. Then the tissue pieces were ground into white powder in a mortar with liquid nitrogen. The tissue powder was mixed with 2 ml ice-cold RIPA lysis buffer and transferred into a 5 ml centrifuge tube. The mixture was placed in an ultrasonic crushing machine on ice for 5 min. The sonication sequential mode was 1 s on and 3 s off, in addition to 30-minute cycles. After the lysis, the mixture was centrifuged at 13,000 g for 30 min at room temperature. The supernatant was collected and a precipitant was added. This mixture was deposited overnight at -20°C. After sedimentation, the sample was centrifuged at 13,000 g for 30 min and the supernatant was removed. The precipitation was washed with 3.6 ml of acetone, then 3.6 ml of anhydrous ethanol, and redissolved in 6 M urea. The concentration of the redissolved protein solution was determined by a bicinchoninic acid (BCA) method (Hussain et al., 2014). The animal experiments were authorized by the Experimental Animal Center of Dalian Medical University.

### Tryptic Digestion of the Protein and Sample Desalination

The above protein solution was diluted to 1 mg/ml with 6 M urea. 1 ml of protein solution was mixed with 50 μL of DTT (200 mM) and incubated at 56°C for 45 min. Then 200 μL of IAA (200 mM) was added and the mixture was placed in dark for 30 min. Then, 7.25 ml of 50 mM NH_4_HCO_3_ aqueous solution and 250 μg trypsin were added in the mixture and incubated at 37°C overnight. Finally, 5 μL FA was added to stop the digestion. Then, the sample was desalted with C18HC packed solid phase extraction microcolumns.

### Enrichment of Glyco- and Phosphopeptides from the Mixture of Fetuin and α-Casein Digest

The enrichment was performed following a reported method (Palmisano et al., 2012b) with minor modification. The tryptic digests of fetuin (5 μg) and α-casein (5 μg) were mixed in 50 μL of 80% ACN/5% TFA, with 1 M glycolic acid (loading buffer). The mixture was added with 1 mg TiO_2_ material and incubated for 15 min. After removal of the supernatant by centrifugation, the TiO_2_ material was washed twice with 50 μL of loading buffer and centrifuged to remove the supernatants. The enriched peptides were used in further experiments.

### On-Line Deglycosylation of the Glycopeptides

The TiO_2_ materials attached with glyco- and phosphopeptides were mixed with 45 μL of 50 mM NH_4_OAc and 5 μL of PNGase F (2,500 U). The resulting solution was incubated for 16 h at 37°C. After centrifugation, the supernatant was collected and desalted for the MS analysis.

### Effect of the Solution pH on the On-Line Deglycosylation

The TiO_2_ materials attached with glyco- and phosphopeptides were separately suspended in four solutions with different pH values: 50 mM HCOONH_4_ (pH 3.0), 50 mM NH_4_OAc (pH 6.9), 50 mM NH_4_HCO_3_ (pH 8.3), and 0.1% NH_3_·H_2_O (v/v, pH 11.5). For each solution, after incubation for 3 h at 37°C, the supernatant was collected by centrifuge and desalted for the MS analysis.

### Effect of the NH_4_OAc Concentration on the On-Line Deglycosylation

The TiO_2_ materials attached with glyco- and phosphopeptides were, respectively, suspended in 45 μL of NH_4_OAc solutions at different concentrations (5, 10, 20, 25, and 50 mM). For each solution, 5 μL of PNGase F (2,500 U) was added and it was incubated at 37°C overnight. After that, the supernatants were removed by centrifugation. The deglycosylated peptides were eluted with 80 μL of 40% ACN/5% TFA. Subsequently, the phosphopeptides were eluted with 80 μL of 5% (v/v) NH_3_·H_2_O. The elution fractions were vacuum-dried and desalted in 19 μL of 50% ACN/0.1% FA. Before the MS analysis, 1 μL internal standard (1 pmol GFB) was added in each sample.

### Effect of the Deglycosylation Time on the On-Line Deglycosylation

6 mg TiO_2_ material, which was attached with glyco- and phosphopeptides, was mixed with 174 μL of 50 mM NH_4_OAc and 5 μL of PNGase F (2,500 U). The mixture was incubated at 37°C and 30 μL of suspended sample was taken after 6, 9, 12, 24, and 48 h incubation, separately. TiO_2_ was isolated by centrifugation, and the deglycosylated peptides and bound phosphopeptides on TiO_2_ were sequentially eluted and treated as described above.

### Optimization of the Deglycosylated Peptides Elution Conditions

Three experiments were performed to investigate the elution effectivities of different eluents. The experiments had the same simultaneous enrichment and on-line deglycosylation processes described above but varied in the later procedures. After on-line deglycosylation, for the first experiment, the 1 mg TiO_2_ material was successively washed with 80 μL of 5 mM NH_4_HCO_3_, 80 μL of 10 mM NH_4_HCO_3_, 80 μL of 20 mM NH_4_HCO_3_, and 80 μL of 5% (v/v) NH_3_·H_2_O. For the second experiment, the 1 mg TiO_2_ material was washed thrice with 20 μL of 50% ACN/1% FA. For the third experiment, the 1 mg TiO_2_ material was successively washed with 120 μL of 40% ACN/5% TFA, 120 μL of 5% TFA, and 80 μL of 5% (v/v) NH_3_·H_2_O. The supernatants of each wash were desalted for the MS analysis.

### Application to the Enrichment of Mouse Brain Actual Sample

**Simultaneous Enrichment:** 100 μg mouse brain lysate was digested by trypsin, desalted, dried, and dissolved in 50 μL of 80% ACN/5% TFA (1 M glycolic acid). The sample was mixed with 2 mg TiO_2_ material and incubated for 30 min. After centrifugation, the precipitate was washed twice with 50 μL of loading buffer. The supernatant was removed by centrifugation.

**On-Line Deglycosylation:** After the simultaneous enrichment, the TiO_2_ material was mixed with 49 μL of 50 mM NH_4_OAc and 1 μL of PNGase F (500 U). The mixture was incubated at 37°C for 12 h. The supernatant was removed by centrifugation.

**Sequential Elution:** The deglycosylated peptides and the bound phosphopeptides on TiO_2_ were sequentially eluted with 80 μL of 40% ACN/5% TFA and 80 μL of 5% (v/v) NH_3_·H_2_O. The eluates were desalted and dried for the MS analysis.

The control experiment was performed on another 100 μg mouse brain lysate according to literature (Palmisano et al., 2012b).

### Mass Spectrometry Analysis and Data Processing

The Dionex UltiMate 3000 RSLC system for chromatographic separation included a C18 trap column (75 μm × 20 mm, 3 μm) and a C18 analytical column (75 μm × 50 mm, 2 μm). The injected volume was 9 μL at a flow rate of 300 nL/min. The mobile phase was as follows: phase A was 0.1% FA and phase B was 80% ACN/0.1% FA. The gradient elution was as follows: 1–4% B, 4 min; 4%–8% B, 2 min; 8%–32% B, 104 min; and 32–90% B, 7 min.

The Orbitrap Eclipse Tribrid mass spectrometer was set as follows. For MS1, the spray voltage was 2.1 kV and the capillary temperature of the ion transport was 320°C. The first-stage full scanning range of mass spectrometry was m/z 300–1,500 with a resolution of 120,000. The RF Lens was set at 40%, the AGQ target was set at 300%, and the maximum injection time (MaxIT) was set to 50 ms. For MS2, the resolution was set at 30,000, the AGQ target was set at 100%, the MaxIT was set to 80 ms, the dynamic exclusion was set to 45 s, the isolation window was set to 1.6 Da, the collision energy was set at 30% HCD, and the fixed first mass was fixed to m/z 110. The data-dependent MS/MS was top speed mode with a cycle time of 2 s. The number of microscans to be performed was set at 1 scan s^−1^.

All the MS raw data were processed by Proteome Discoverer and searched with SEQUEST against the mouse proteins in the UniProt database. The trypsin cleavage with a maximum of two leakage sites was allowed. Carbamidomethyl (C) was set as a fixed modification, and oxidation on methionine (M), acetylation of protein N terminus, phospho-modification (STY), and deamination (N) were set as the variable modifications. The false discovery rate (FDR) was set at 1%. The other conditions were set by default.

## Results and Discussion

### The Workflow of the Simultaneous Enrichment, On-Line Deglycosylation, and Sequential Elution Strategy to Analyze Glyco- and Phosphopeptides

In this work, we developed a new strategy to simultaneously enrich and sequentially separate glyco- and phosphopeptides with high efficiency and recovery. Firstly, TiO_2_ is used to simultaneously enrich glyco- and phosphopeptides from a complex sample, which are bound to TiO_2_ with their glycan chains or phosphate groups, respectively (Scheme 1B). After the enrichment, unmodified peptides are removed, and glyco- and phosphopeptides are attached on TiO_2_. Secondly, the on-line deglycosylation using PNGase F is carried out to remove the glycans from the glycopeptides and produce deglycosylated peptides. Thus, the deglycosylated peptides are released from TiO_2_ and collected. Thirdly, the attached phosphopeptides on TiO_2_ are eluted. Finally, the deglycosylated peptides and the phosphopeptides are characterized with MS, respectively.

### Effect of Solution pH on On-Line Deglycosylation

**FIGURE 1:**
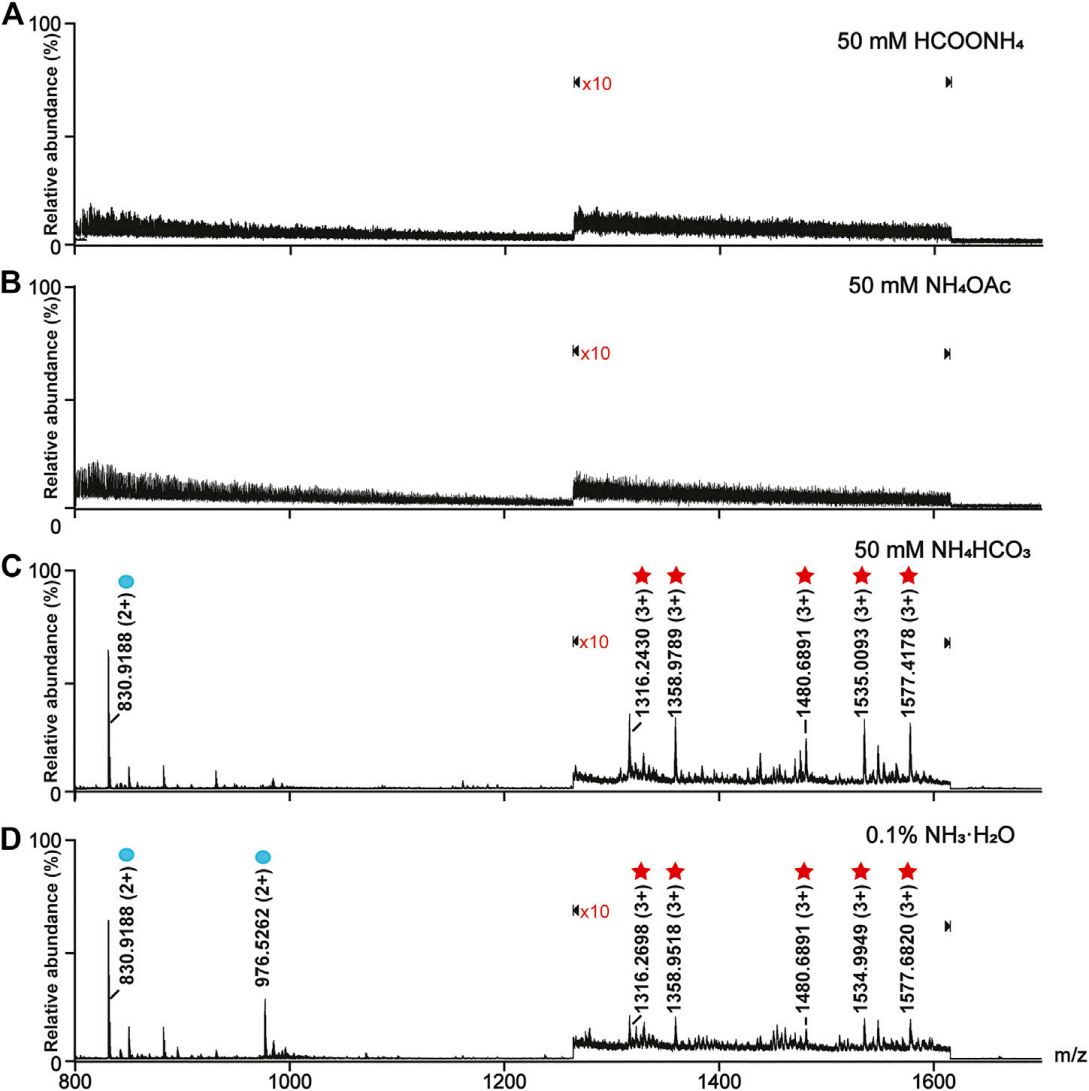
Optimization of the online deglycosylation condition in different solution pH. **(A)** 50 mM HCOONH_4_ (pH 4.6), **(B)** 50 mM NH_4_OAc (pH 6.9), **(C)** 50 mM NH_4_HCO_3_ (pH 8.3), and **(D)** 0.1% (v/v) NH_3_·H_2_O (pH 11.5). The glycopeptides and phosphopeptides were marked with red stars and blue circles, respectively. The signals at m/z ranging from 1,265 to 1,615 in the mass spectra were amplified tenfold (×10), where “×10” represents the magnification times.

The solution pH was an important parameter for the on-line deglycosylation. An ideal solution pH should not only work out for the deglycosylation but also have no impact on the phosphopeptide retention on TiO_2_. Here we investigated the effect of the solution pH on the glyco- and phosphopeptides retention on TiO_2_ (Figure 1). After the enrichment, the TiO_2_ materials bound with PTM-peptides were separately resuspended in four solutions: 50 mM HCOONH_4_ (pH 4.6), 50 mM NH_4_OAc (pH 6.9), 50 mM NH_4_HCO_3_ (pH 8.3), and 0.1% NH_3_·H_2_O (v/v, pH 11.5). After incubation for 3 h and centrifugation, the supernatants were collected, desalted, and analyzed with MS. As shown in Figures 1A,B, neither glycopeptides nor phosphopeptides can be detected in the mass spectra of the HCOONH_4_ or NH_4_OAc treated samples. On the contrary, both glycopeptides (marked with red stars) and phosphopeptides (marked with blue circles) are clearly observed in the mass spectra of the NH_4_HCO_3_ and NH_3_·H_2_O treated samples (Figures 1C,D). These results implied that HCOONH_4_ and NH_4_OAc met the basic requirement for the on-line deglycosylation. Considering that the pH value of the NH_4_OAc solution (pH 6.9) was much closer to the reaction condition pH (pH 7.5) of the PNGase F deglycosylation, this solution pH was chosen in later experiments.

### Effect of the NH_4_OAc Concentration on the On-Line Deglycosylation

**FIGURE 2:**
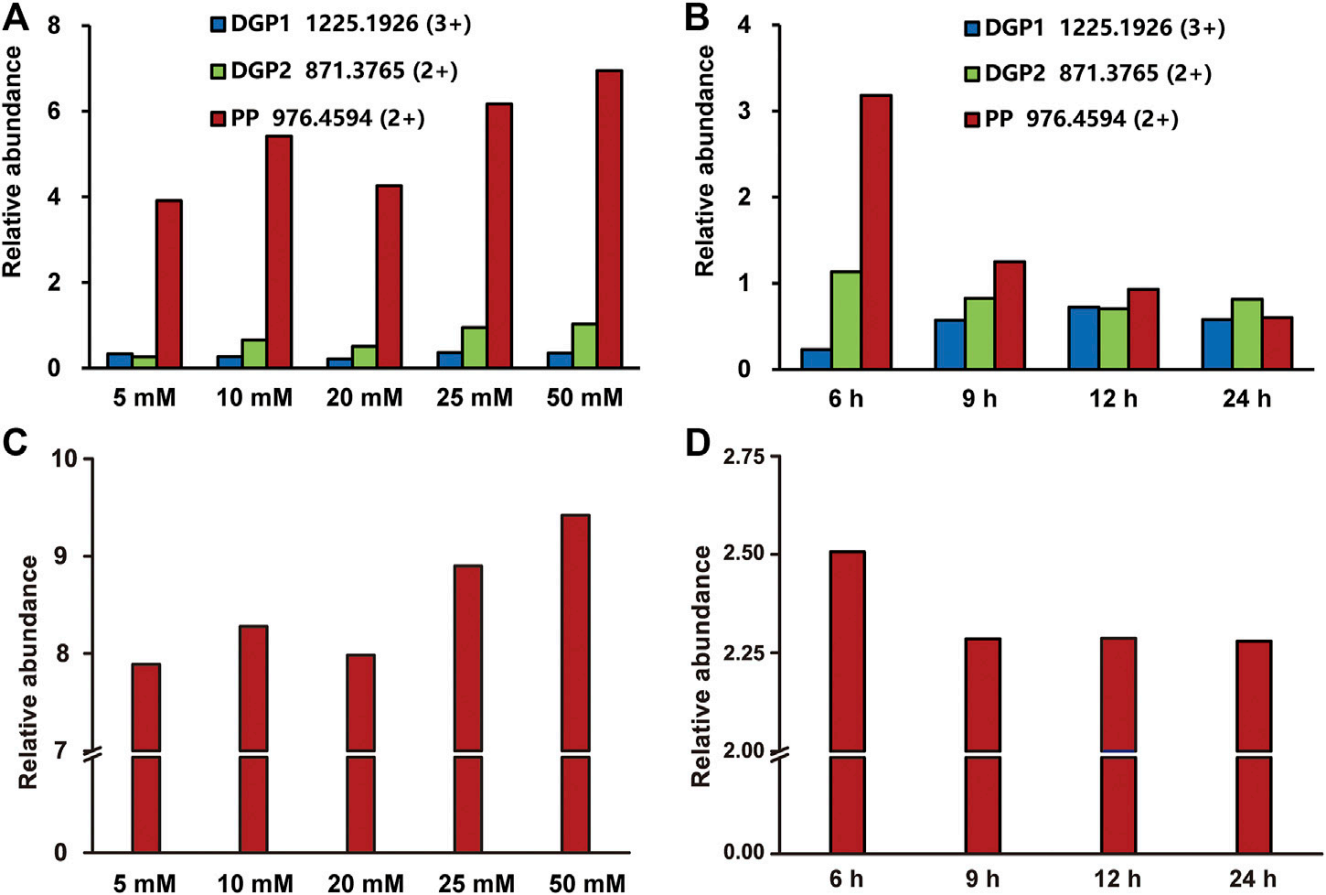
Optimization of the on-line deglycosylation conditions in different NH_4_OAc concentrations **(A, C)** and deglycosylation times **(B, D)**. The relative abundances of the target deglycosylated peptide and the phosphopeptide obtained after the online deglycosylation **(A)** in different concentrations of NH_4_OAc or **(B)** for different deglycosylation times. The relative abundances of the **s**tandard phosphopeptide **(C)** in different concentrations of NH_4_OAc or **(D)** in 50 mM NH_4_OAc for different times. DGP, deglycosylated glycopeptide; PP, phosphopeptide.

The buffer concentrations have an impact on the deglycosylation efficiency by influencing the proton exchange during the enzyme catalysis (Cheison et al., 2011; Liu et al., 2009). Thus, the effect of the NH_4_OAc concentration on the efficiency of the on-line deglycosylation was further investigated. We measured the relative abundances of the deglycosylated peptides and phosphopeptide in the mass spectra of the elutes after the on-line deglycosylation with NH_4_OAc in five different concentrations (5, 10, 20, 25, and 50 mM). The deglycosylated peptides at m/z 1,225.1926 (3+) and m/z 871.3765 (2+) and the phosphopeptide at m/z 976.4594 (2+) were chosen as targets of interest. The relative abundances of the target peptides were quantified with GFB as an internal standard. As shown in Figure 2A, the relative abundances of the deglycosylated peptide and the phosphopeptide gradually increased with the NH_4_OAc concentration increasing and reached the maximum in 50 mM NH_4_OAc during the investigated concentration range. For the deglycosylated peptide, this phenomenon might be attributed to the improved efficiency of proton exchange between PNGase F and the glycopeptides with increased concentrations of NH_4_OAc, which is consistent with the reported result (Cheison et al., 2011). On the other hand, the enhanced relative abundance of the phosphopeptide might result from the reduced degree of its hydrolysis with the increased NH_4_OAc concentration. In order to further correlate the relationship between the hydrolysis degree of the phosphopeptide and the NH_4_OAc concentration, the relative abundances of the standard phosphopeptide in different concentrations of the NH_4_OAc solution were tested (Figure 2C). When the NH_4_OAc concentration was low, the relative abundance of the standard phosphopeptide was low, which was ascribed to the high hydrolysis degree of the standard phosphopeptide. As the NH_4_OAc concentrations increased, the relative abundance of the standard phosphopeptide gradually increased, which was attributed to the decreased hydrolysis degrees of the standard phosphopeptide. It seemed that higher NH_4_OAc concentrations were favorable for inhibiting the phosphopeptide hydrolysis. These results were in good agreement with that of Figure 2A. Taken together, 50 mM NH_4_OAc was chosen for further on-line deglycosylation.

### Effect of the Deglycosylation Time on the On-Line Deglycosylation

Compared with the glycopeptides, the phosphopeptides are more susceptible to external influence and are unstable (Hu et al., 2020). During the deglycosylation process, the phosphopeptides hydrolyze as time goes on, and, therefore, the deglycosylation time was another important factor for the on-line deglycosylation. In order to optimize the deglycosylation time, we measured the relative abundances of the target deglycosylated peptide and the phosphopeptide after the on-line deglycosylation with different times. As shown in Figure 2B, the relative abundance of the deglycosylated peptide at m/z 1,225.1926 (3+) increased over time until 12 h was reached, in sharp contrast to the relative abundance of the phosphopeptide which gradually decreased. The latter might result from the hydrolysis of the phosphopeptide (Figure 2D). Considering both the phosphopeptide hydrolysis degree and the glycopeptide deglycosylation efficiency, 12 h was chosen as further on-line deglycosylation time.

### Optimization of the Elution Conditions for the Deglycosylated Peptides

**FIGURE 3:**
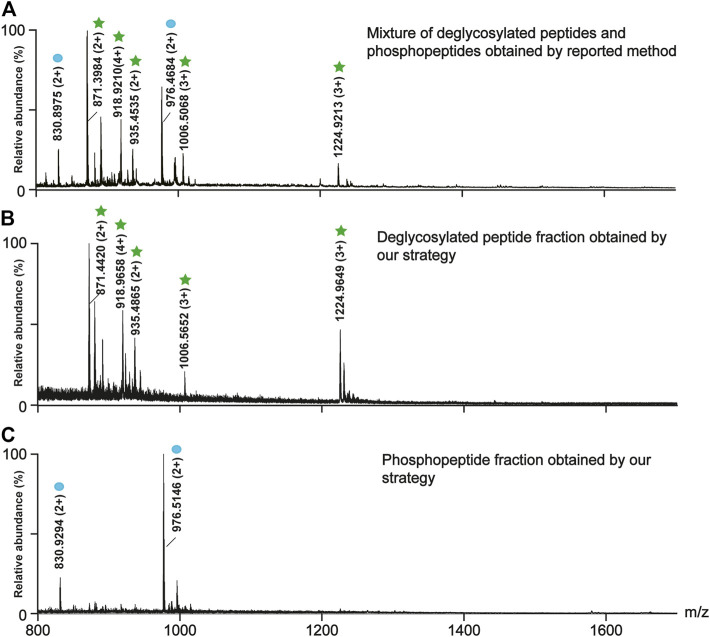
Comparison between the reported method **(A)** and our strategy in the separation efficiency of the deglycosylated peptides **(B)** and the phosphopeptides **(C)**. **(A)** The mass spectra of the mixture of the deglycosylated peptides and the phosphopeptides obtained by the reported method. The mass spectra of **(B)** the deglycosylated peptide fraction and **(C)** the phosphopeptide fraction obtained by our strategy. The deglycosylated peptides and the phosphopeptides are marked with green stars and blue circles, respectively.

After the on-line deglycosylation, the released deglycosylated peptides were re-adsorbed on TiO_2_, which is line with the previous studies that non-modified peptides tend to be nonspecifically adsorbed on TiO_2_ due to Lewis acid-base interaction between the carboxyl groups on the peptide chains and TiO_2_ and the hydrophobic interaction between the hydrophobic peptide chains and TiO_2_ (Palmisano et al., 2012b). To efficiently elute and collect the absorbed deglycosylated peptides but not the phosphopeptides, the elution efficiencies of three types of eluents were evaluated. The three types of eluents were NH_4_HCO_3_ solutions with different concentrations (5, 10, and 20 mM), 50% ACN/1% FA, and 40% ACN/5% TFA. As shown in Supplementary Figure 1, 5 and 10 mM NH_4_HCO_3_ could not elute any PTM-peptides, while 20 mM NH_4_HCO_3_ could co-elute the deglycosylated peptides and the phosphopeptides. Therefore, NH_4_HCO_3_ was not suitable for the deglycosylated peptides elution. As to the acidic conditions, after the elution with 50% ACN/1% FA, the deglycosylated peptides were rarely observed (Supplementary Figure 2), while all the targeted deglycosylated peptides but not the phosphopeptides could be detected in the eluate of 40% ACN/5% TFA (Figure 3B). Afterward, the bound phosphopeptides on TiO_2_ were found from the eluate of 5% (v/v) NH_3_·H_2_O (Figure 3C), which was consistent with the phosphopeptides obtained by using the reported simultaneous enrichment, the co-elution, and the deglycosylation method (Figure 3A). Thus, 40% ACN/5% TFA was used as the eluent of the deglycosylated peptides.

### Analysis of Glyco- and Phosphopeptides from the Mouse Brain

**FIGURE 4:**
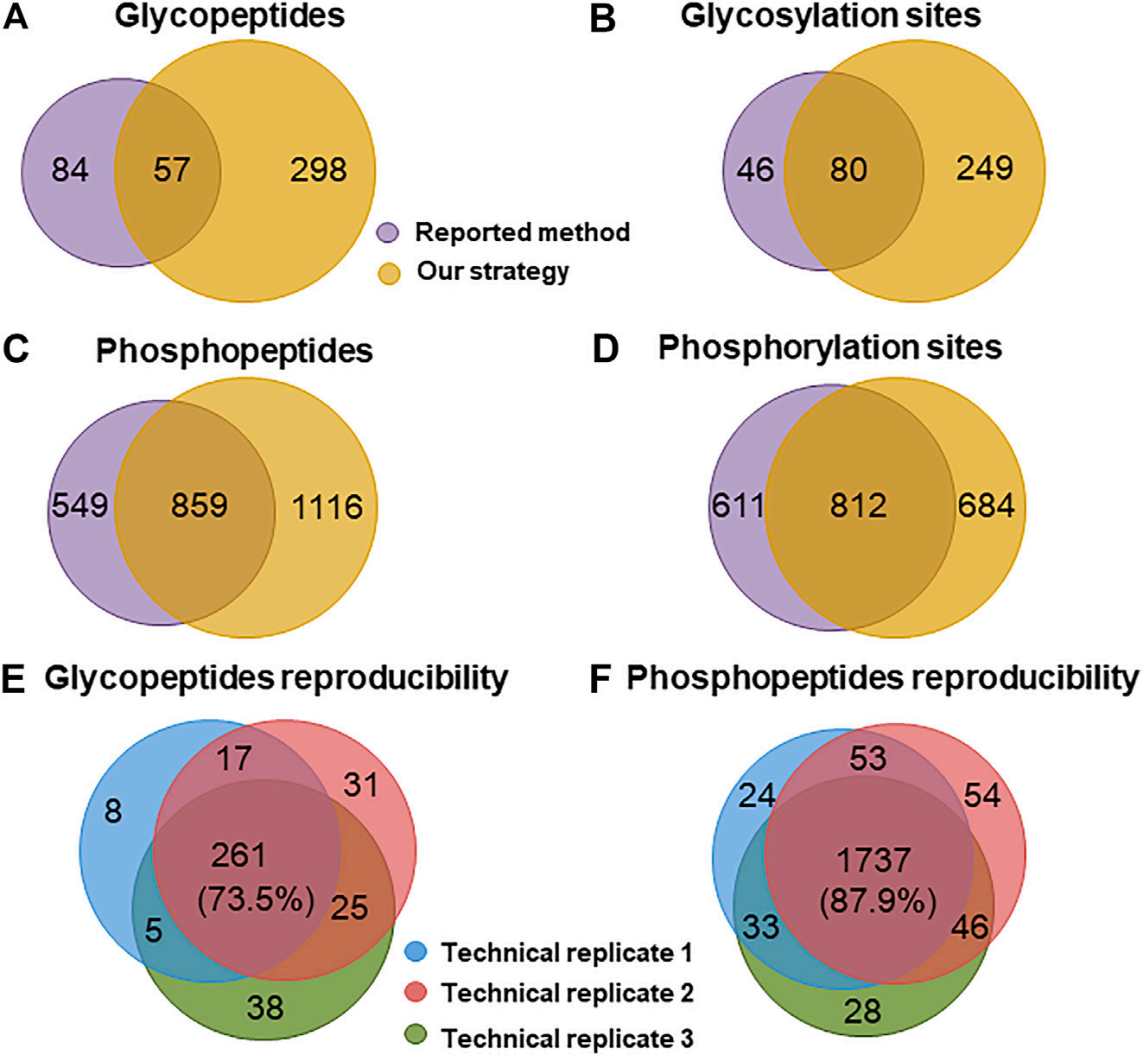
Comparison of the performance for identifying glyco- and phosphopeptides from a mouse brain. Venn diagram analysis of the number of **(A)** glycopeptides, **(B)** glycosylation sites, **(C)** phosphopeptides, and **(D)** phosphorylation sites identified with the reported method and our strategy. Reproducibility for **(E)** glyco- and **(F)** phosphopeptides among three technical replicates of our strategy.

To examine the effectiveness of our strategy, we applied it to analyze glyco- and phosphopeptides from a 100 μg mouse brain. Meanwhile, the reported method (Palmisano et al., 2012b) was carried out for comparison. By using our strategy, a total of 329 glycosylation sites (Supplementary Table 1) from 355 glycopeptides (Supplementary Table 2) and 1,496 phosphorylation sites (Supplementary Table 3) from 1975 phosphopeptides (Supplementary Table 4) were identified from three technical replicates. In sharp contrast to these, only 126 glycosylation sites (Supplementary Table 5) from 141 glycopeptides (Supplementary Table 6) and 1,423 phosphorylation sites (Supplementary Table 7) from 1,408 phosphopeptides (Supplementary Table 8) were identified from the identical sample using the reported method (Figures 4A–D). The numbers of the identified glyco- and phosphopeptides with our strategy were 2.5 and 1.4 folds of those with the reported method, respectively. Moreover, our strategy demonstrated high reproducibility with 73.5 and 87.9% common glyco- and phosphopeptides among three technical replicates (Figures 4E,F), respectively. The reproducibility between two technical replicates was much higher, 77.0 ± 2.2% and 91.8 ± 0.3% for glyco- and phosphopeptides, respectively. Besides the high reproducibility, the numbers of the phosphopeptides in the deglycosylated glycopeptide fraction and the phosphopeptide fraction were 67 and 2,298, respectively, suggesting a low degree of overlap between the deglycosylated glycopeptide and the phosphopeptide fraction in our strategy.

**FIGURE 5:**
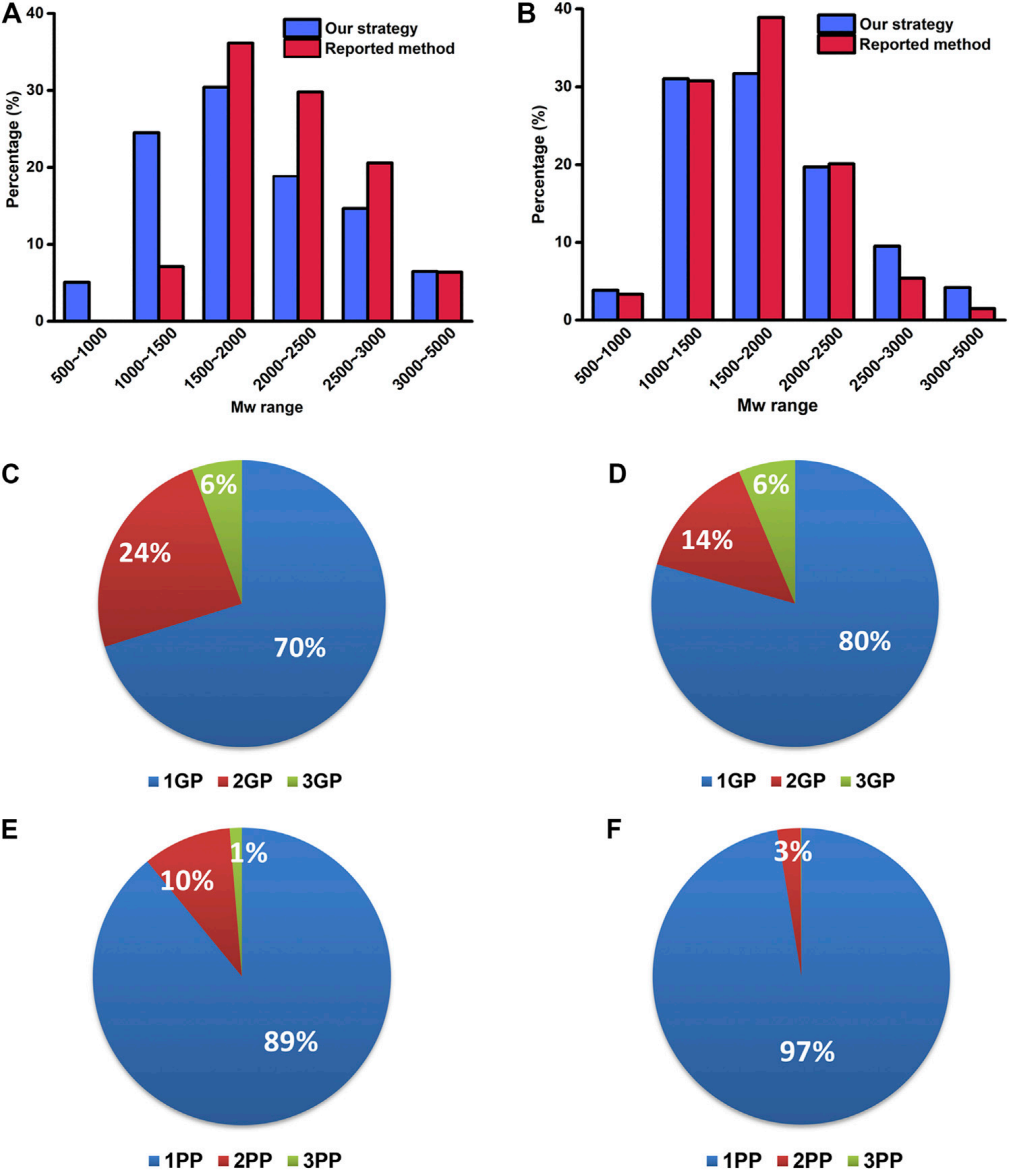
The Mw distribution of identified glyco- **(A)** and phosphopeptides **(B)** by our strategy and the reported method. The percentages of the identified PTM-peptides with single glycosylation/phosphorylation site and multiple glycosylation/phosphorylation sites by our strategy **(C, E)** and the reported method **(D, F)**. 1–3 GP: glycopeptide with 1–3 glycosylation sites; 1–3 PP: phosphopeptide with 1–3 phosphorylation sites.

The property differences of the identified PTM-peptides between the reported method and our strategy were further investigated. The molecular weight (Mw) distribution of the identified PTM-peptides and the percentages of the peptides with single-PTM and multiple PTM sites were compared (Figure 5).

As shown in Figure 5A, the Mw distribution pattern of the identified glycopeptides is consistent between the reported method and our strategy in a higher Mw range (1,500–5,000 Da) is consistent between the reported method and our strategy. However, in the lower Mw range of 500–1,000 Da and 1,000–1,500 Da, the number of the identified glycopeptides with our strategy accounts for 5.1 and 24.5% of the total ones, respectively, in sharp contrast to that of 0 and 7.1% with the reported method. These results indicate that our strategy has advantages in the enrichment and identification of low Mw glycopeptides. This result might be ascribed to the fact that our strategy omits the desalting procedure after the deglycosylation and retains well the low Mw glycopeptides.

As to the percentages of the peptides with single-PTM and multiple PTM sites, the number of identified glycopeptides with two glycosylation sites with our strategy accounts for 24% of the total glycopeptides, compared with only 14% with the reported method (Figures 5C,D). Similarly, the number of identified phosphopeptides with two phosphorylation sites with our strategy accounts for 10% of the total phosphopeptides, in sharp contrast to that of 3% with the reported method (Figures 5E,F). These results revealed the superiority of our strategy in the identification of the peptides with multiple glycosylation/phosphorylation sites. It is possibly because our strategy reduced the complexity of the samples and increased the number of the identified low-abundance peptides with multiple glycosylation/phosphorylation sites.

The above results indicated that our strategy not only realized the sequential elution of glyco- and phosphopeptides but also significantly increased the numbers of identified glyco- and phosphopeptides. Our study provided an effective means for the simultaneous characterization of the protein glycosylation and phosphorylation.

## Conclusion

In this work, we developed a strategy for the analysis of glyco- and phosphopeptides based on the simultaneous enrichment with TiO_2_, the on-line deglycosylation, and the sequential elution. The application of this strategy to the mouse brain tissue achieved a higher number of targeted peptides compared with the reported method. Our strategy shows some advantages in the simultaneous analysis of glyco- and phosphopeptides: 1. The on-line deglycosylation and the sequential elution can separate the deglycosylated peptides and the phosphopeptides into two different fractions, which can reduce the complexity of the samples and improve the coverage of the identified PTM-peptides under the data dependent acquisition (DDA) mode. 2. The reduction of the sample complexity can reduce the ion suppression and increase the number of low-abundance glyco- and phosphopeptides with multiple glycosylation and phosphorylation sites. 3. The elimination of the desalting procedure after the deglycosylation can reduce the loss of low Mw glycopeptides. 4. A neutral online deglycosylation condition can effectively inhibit the hydrolysis of the phosphopeptides and increase the number of identified phosphopeptides.

To sum up, this work will provide a new idea to expand the applications of TiO_2_ and tackle the problems in the simultaneous analysis of the protein glycosylation and other multiple PTMs.

## Data Availability

The datasets presented in this study can be found in online repositories. The names of the repository/repositories and accession number(s) can be found in the article/Supplementary Material.
