# Supplementary material for: TiO2 Simultaneous Enrichment, On-Line Deglycosylation, and Sequential Analysis of Glyco- and Phosphopeptides
Source: Front Chem. 2021 Aug 11;9:703176. doi: 10.3389/fchem.2021.703176 (PMC8385670; doi:10.3389/fchem.2021.703176)
Supplement: Supplementary file 4 [file DataSheet1.pdf]

Supplementary Information

TiO$_2$ Simultaneous Enrichment, On-Line Deglycosylation, and Sequential Analysis of Glyco- and Phosphopeptides

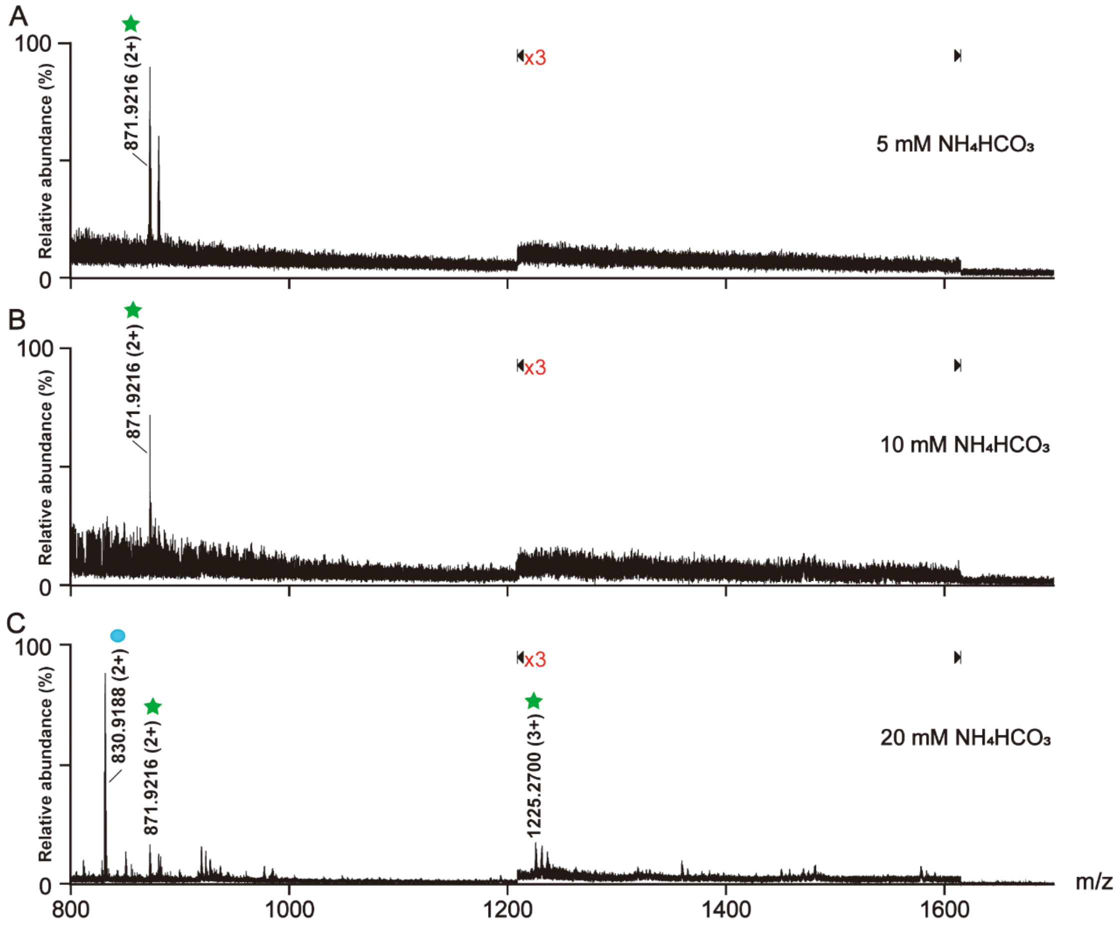

Supplementary Figure 1 Mass spectra of PTM-peptides obtained after TiO$_2$ simultaneous enrichment, on-line deglycosylation and elution with (A) 5 mM NH_4HCO_3; (B) 10 mM NH_4HCO_3 and (C) 20 mM NH_4HCO_3. Glycopeptides, deglycosylated peptides and phosphopeptides are marked with red stars, green stars and blue circles. The signals at m/z ranged from

1208 to 1613 in the mass spectra were amplified by 3-fold (×3), where “×3” represents the magnification times.

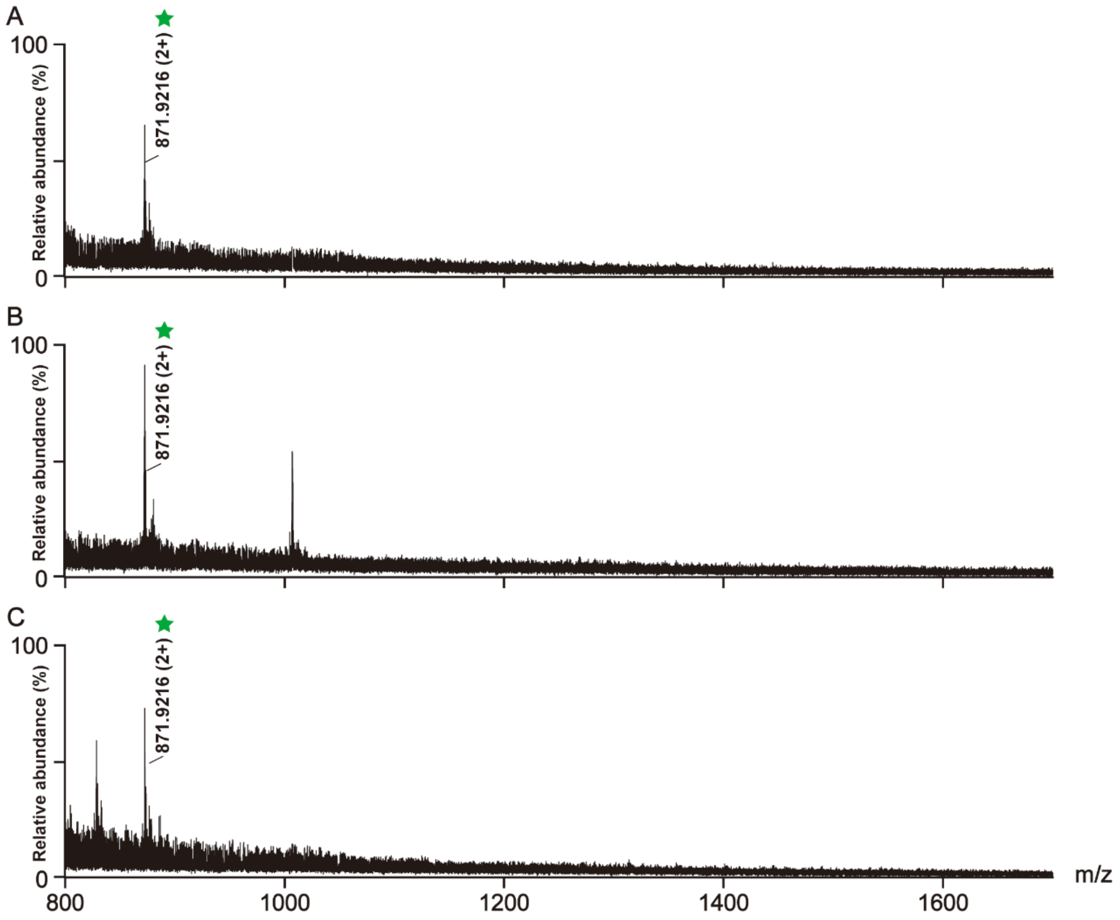

Supplementary Figure 2 Mass spectra of PTM-peptides obtained after TiO_2 simultaneous enrichment, on-line deglycosylation and elution thrice with (A-C) 50% ACN/ 1% FA. Deglycosylated peptides are marked with green stars.
